# Supplementary material for: Exploring healthcare professionals’ beliefs, experiences and opinions of family-centred conversations when a parent has a serious illness: A qualitative study
Source: PLoS One. 2022 Nov 28;17(11):e0278124. doi: 10.1371/journal.pone.0278124 (PMC9704560; doi:10.1371/journal.pone.0278124)
Supplement: Attachment — Submitted filename: Response to reviewers comments.docx [file pone.0278124.s002.docx]

**Exploring healthcare professionals’ beliefs, experiences and opinions of family-centred conversations when a parent has a serious illness: a qualitative study**

**Response to reviewer’s comments**

We are grateful to the reviewers for considering our revised manuscript and are delighted that they are happy with our revisions.

**Reviewer 1:**

Thank you very much for your helpful information regarding Braun and Clarke’s methodology and will be sure to take this into account in our future work.

**Reviewer 2:**

Thank you for highlighting the importance of religious and spiritual beliefs and the parallels with talking to patients about their family network. We have added the suggested reference of Sparaco *et al*., 2022, as well as a systematic review regarding this important literature (Best *et al.,* 2016)
